# The technological assessment of green buildings using artificial neural networks

**DOI:** 10.1016/j.heliyon.2024.e36400

**Published:** 2024-08-15

**Authors:** Ying Huang

**Affiliations:** a College of Art & Design, Putian University, Fujian, China; b Design Innovation Research Center of Humanities and Social Sciences Research Base of Colleges and Universities in Fujian Province, Fuzhou, China

**Keywords:** Green buildings, Internet of things, Artificial neural network, Technological assessment, Environmental monitoring

## Abstract

This study aims to construct a comprehensive evaluation model for efficiently assessing appropriate technologies within green buildings. Initially, an Internet of Things (IoT)-based environmental monitoring system is devised and implemented to collect real-time environmental parameters both inside and outside the building. To evaluate the technical suitability of green buildings, this study employs a multifaceted approach encompassing various criteria, including energy efficiency, environmental impact, economic benefits, user comfort, and sustainability. Specifically, it involves real-time monitoring of environmental parameters, analysis of energy consumption data, and indoor environmental quality indicators derived from user satisfaction surveys. Subsequently, a Multi-Layer Perceptron (MLP) is selected as a conventional artificial neural network (ANN) model, while a Long Short-Term Memory (LSTM) model is chosen as an advanced recurrent neural network model in the realm of deep learning. These models are utilized to process and explore the collected data and assess the technical suitability of green buildings. The dataset comprises physical quantities such as temperature, humidity, and light intensity, as well as economic indicators including energy efficiency and building operating costs. Furthermore, the assessment process considers the building's life cycle assessment and indoor environmental quality factors such as health, comfort, and safety. By incorporating these comprehensive criteria, a holistic evaluation of green building technologies is achieved, ensuring the selected technologies' suitability and effectiveness. The model prediction results demonstrate that the proposed hybrid evaluation model exhibits high accuracy and robust stability in predicting building environmental parameters. For instance, the Root Mean Square Error (RMSE) for temperature prediction is 1.2 °C, the Mean Absolute Error (MAE) is 0.9 °C, and the determination coefficient (R^2^) reaches 0.95. Similarly, for humidity prediction, the RMSE, MAE, and R^2^ are 3.5 %, 2.8 %, and 0.88. Compared to the traditional MLP and LSTM models alone, the proposed hybrid model shows significant improvements in predicting building energy consumption, with approximately 15 % and 12 % reductions in RMSE and MAE, respectively, and an increase in R^2^ values of approximately 7 percentage points. These findings indicate that by amalgamation of the IoT and ANNs, this study successfully establishes a comprehensive model for accurately assessing technologies suitable for green buildings. This approach offers a novel perspective and methodology for the design and evaluation of green buildings.

## 1 Introduction

### 1.1 Research background and motivations

The integration of Internet of Things (IoT) technology into the domain of green buildings is witnessing an escalating trajectory [1]. By amalgamating sensor networks, wireless communication technology, and big data analytics, IoT has realized real-time monitoring and remote management of pivotal metrics like indoor environmental quality, energy consumption, and water resource utilization within green buildings [[2], [3], [4]]. For instance, smart lighting systems autonomously modulate brightness levels based on human activity and natural light conditions, while building energy management systems furnish real-time feedback and optimize energy distribution, thus curtailing unnecessary losses [[5], [6], [7]]. Nevertheless, much of the extensive data collected by IoT technology remains underutilized and unexplored [8].

Concurrently, artificial neural networks (ANNs) have proven their value across diverse engineering and technical domains due to their robust learning capabilities and advantages in nonlinear modeling [[9], [10], [11]]. Within green building research, ANNs can simulate intricate physical processes within buildings, such as heat and moisture transfer, light variations, and air quality dynamics. This capability enables the quantitative prediction and optimization of building performance [12]. Moreover, by assimilating knowledge from precedent cases, they can uncover synergies and potential bottlenecks among various green building technologies, providing a more scientific and refined basis for technology selection [[13], [14], [15]].

However, prevailing assessment methodologies for identifying suitable green building technologies predominantly rely on conventional static evaluation frameworks. These methodologies often fail to capture the dynamic attributes of building performance across temporal and spatial dimensions, missing out on the full potential of contemporary information technology [16]. Furthermore, these methodologies frequently encounter challenges in managing interactions among myriad variables and grappling with uncertainties, which impedes their ability to meet the increasing demands for intelligence and customization in green building advancement [[17], [18], [19]].

In recent years, progress has been made in assessing the technical suitability of green buildings, yet notable shortcomings persist. Previous research has typically focused on individual technology performance or the impact of singular environmental factors on overall building performance. This approach often lacks a comprehensive model that thoroughly analyzes and predicts the building's environmental, energy, and economic impacts. It overlooks potential synergies from integrating multiple technologies and the intricate dynamic relationships among various environmental parameters. Many scholars have attempted to address this issue using traditional statistical analysis or simplistic predictive models. For instance, Ravichandran et al. (2024) employed a linear regression model to assess the influence of solar panel energy efficiency on total building energy consumption [20]. However, these methodologies often struggle to handle inherent nonlinear relationships and temporal dependencies in the data, leading to limited accuracy and reliability of assessment outcomes. Moreover, while some studies have explored the application of IoT technology for real-time environmental monitoring, they have generally underutilized the vast amount of collected data for optimizing building performance.

For instance, Zhu et al. (2022) developed an IoT-based system to monitor CO_2_ levels within buildings [21], yet did not delve into how this data could comprehensively evaluate environmental quality and user comfort in buildings. Farghaly et al. (2020) [22] proposed an effective hybrid feature selection method, which was a filter-based method. It provided scores for each feature and then automatically specified thresholds based on the dataset being used to select an important feature subset to construct the model, thereby reducing the required execution time and memory.

Given these deficiencies and research gaps, this study poses a novel question: How can a comprehensive evaluation model be developed to monitor and analyze the real-time environmental and energy performance of green buildings while also accounting for the synergistic effects of technology integration and user comfort?

To address this challenge, the study proposes a comprehensive evaluation model that integrates IoT and ANNs, particularly leveraging Multi-Layer Perceptron (MLP) and Long Short-Term Memory (LSTM) network architectures. The model aims to achieve the following objectives:

1) Real-time collection and analysis of environmental parameters, such as temperature, humidity, and light intensity, both inside and outside buildings.
2) Holistic evaluation of the energy efficiency performance of technologies, considering their implications on environmental impact, economic viability, user comfort, and sustainability.
3) Utilization of deep learning (DL) techniques to uncover nonlinear relationships and time series dependencies within the data, thereby enhancing prediction accuracy and stability.

Through this approach, the existing research gaps are bridged, providing a more comprehensive and scientifically grounded framework for the design and assessment of green buildings. Furthermore, this proposed model may offer valuable insights for technology assessment across diverse domains beyond construction.

### 1.2 Research objectives

This study devises a new methodology for precisely evaluating the efficacy of green building technologies. The experiment integrates the real-time data collection capability of IoT with the robust data analysis and predictive capabilities of ANNs. This fusion yields a comprehensive system tailored for the automated and intelligent assessment of appropriate technologies within green buildings. A selection of representative green building projects is enlisted to validate the proposed method's efficacy and superiority in evaluating green building technologies via integrating IoT and ANNs. These projects undergo empirical scrutiny employing both conventional assessment techniques and the novel approach.

## 2 Literature review

In recent years, the significance of IoT technology in the green building field has escalated. Nižetić et al. (2020) and Ahmad & Zhang (2021) highlighted the pivotal role of IoT in real-time data acquisition of vital information such as internal environmental parameters, energy consumption data, and equipment operating conditions, facilitated by intelligent sensors and wireless communication networks [[23], [24], [25]]. Concurrently, ANNs demonstrated myriad successful applications in simulating and predicting building performance. Chen et al. (2023) and Yang et al. (2023) showcased the effective utilization of ANNs in assessing building energy consumption, comfort levels, and structural health monitoring, offering decision support across various stages of the building lifecycle [26,27]. However, most extant studies predominantly focused on optimizing the performance of individual systems or components. The exploration of comprehensive performance evaluation and optimization strategies is limited considering the interactions among diverse green building technologies. Present research about evaluating suitable green building technologies primarily concentrated on establishing qualitative evaluation frameworks and refining quantitative calculation methodologies. Mrówczyńska et al. (2021) experimented with methodologies such as multivariate statistical analysis and fuzzy logic to quantify factors influencing technology selection [28]. Additionally, Al Zihad et al. (2023) highlighted the insufficiency of existing methodologies in accounting for dynamic changes in building usage environments, user behavior patterns, and the interplay between technologies. Thus, it posed challenges in accurately predicting the green building technologies’ long-term performance and energy efficiency in practical applications [29]. Djoulde et al. (2024) [30] used color filter array images to classify pepper seeds around the building. The most successful model was the Support Vector Machine (SVM), which reached an accuracy of 0.87, a precision of 0.874, a recall of 0.873, and an F1 score of 0.874. Dtissibe et al. (2024) [31] designed and compared the performance of machine learning and DL-based models, such as one-dimensional convolutional neural networks (CNNs), LSTM, and MLP. Moreover, they developed effective and efficient flood risk management policies to make the far North region of Cameroon more resilient to flood crises. Shams et al.(2024) [32] introduced a novel DL model called the self-attentional layer in CNNs, specifically designed to detect acoustic data from a broad dataset containing emergency vehicle alarms and road noise.

This study seeks to address the deficiencies observed in previous research, including the lack of real-time data, limitations of DL models in forecasting building performance, and constraints on the comprehensive evaluation of suitable green building technologies in intricate environments. The study deeply integrates the advantages of IoT technology and ANNs to surmount these challenges. By constructing an intelligent evaluation framework that leverages self-learning and adaptability, the objective is to facilitate dynamic monitoring, precise assessment, and optimization recommendations for green building technologies during operational phases. This study is anticipated to furnish more dependable technical support and decision-making criteria, thus fostering sustainable green building industry development and technological innovation.

## 3 Research methodology

### 3.1 IoT system and environmental monitoring

**Fig. 1:**
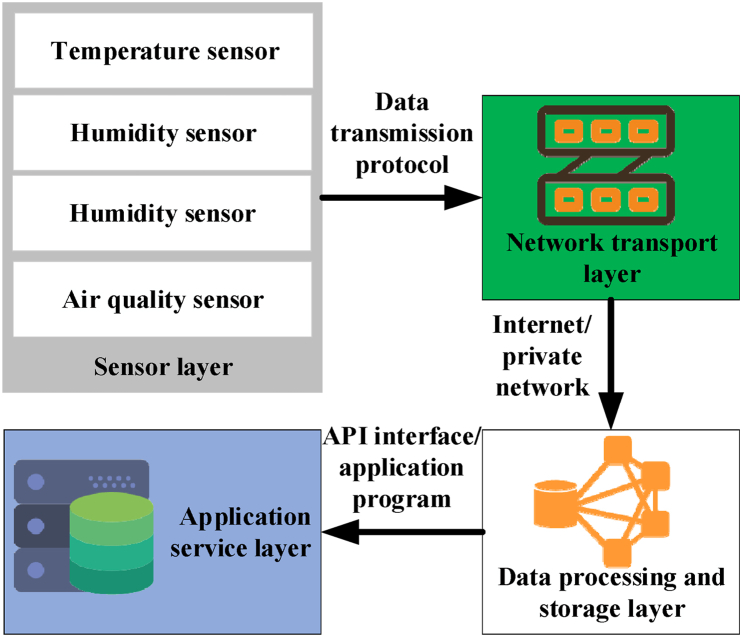
Architecture of the IoT system.

Incorporating an IoT system in green buildings can realize efficient monitoring and intelligent management of diverse environmental parameters, thereby augmenting building efficiency and optimizing indoor environmental quality [[33], [34], [35]]. This study delineates the architecture of the IoT system for environmental monitoring [36], with the specific structure depicted in Fig. 1.

In Fig. 1, the sensor layer primarily encompasses various environmental monitoring sensors, such as temperature sensors, humidity sensors, air quality detectors, and light intensity sensors. These sensors are strategically deployed across different building areas, facilitating the collection of real-time environmental indicator data. Moving to the network transmission layer, data gathered by sensors are transmitted to gateway devices via wireless communication technology and subsequently uploaded to cloud servers through the internet [[37], [38], [39]]. The data processing and storage layer is managed by the cloud server, which receives and stores all sensor data. It conducts preliminary data preprocessing tasks such as integrating time series data, cleaning data, and eliminating redundancy. Transitioning to the application service layer, a monitoring platform is established, enabling administrators to access real-time environmental data from various monitoring points. Additionally, algorithmic analysis is employed to discern trends, while automatic adjustment and alert mechanisms are implemented [[40], [41], [42]].

To facilitate environmental monitoring, the IoT system continually collects and updates environmental parameter values, ensuring the data's timeliness. Integrated data from diverse sources can be leveraged to establish building performance models, thus facilitating energy consumption simulations and developing energy-saving strategies. Moreover, real-time monitoring data triggers automated control logic, which adjusts the operational status of building facilities. For instance, it dynamically regulates the air conditioning system's output in response to fluctuations in room temperature. In essence, integrating IoT systems in green buildings substantially enhances the accuracy and efficiency of environmental monitoring, thereby furnishing robust technical support for intelligent management and optimization in green building practices.

### 3.2 Selection of ANNs models and application adaptability

In green building assessments, selecting the appropriate neural network model is paramount for capturing intricate data relationships and forecasting building performance indicators amidst varying influencing factors [[43], [44], [45]]. This study chooses MLP and LSTM based on several considerations. In this study, the choice of MLP and LSTM as modeling methods is substantiated by several factors. Firstly, MLP, with its multi-layer neural structure and nonlinear activation function, proficiently models and learns complex nonlinear data relationships. Within green building assessment contexts, it is particularly effective for processing static or quasi-static attribute data without explicit time-series components, such as building physical characteristics, design parameters, and geographical location information. MLP's capability to perform multi-level feature extraction and abstraction helps reveal hidden interactions and influences among various building attributes, facilitating precise performance assessments. Secondly, LSTM, a recurrent neural network (RNN) structure tailored for sequence data processing, excels at capturing and processing long-term dependencies in time series. In green building environment monitoring, numerous key parameters like energy consumption and indoor environmental quality (temperature, humidity, light intensity, etc.), exhibit dynamic changes over time. LSTM effectively leverages this time-series data to analyze and forecast evolving trends of building environment parameters in future periods and assess the implications for building performance and technology selection. The intrinsic advantages of MLP and LSTM make them well-suited for this study's modeling needs. MLP efficiently handles static attribute data and extracts critical high-level abstract features essential for building performance evaluation. However, LSTM processes dynamic time series data to capture and predict variations in building environment parameters over time. By integrating these two models comprehensively, the study introduces a hybrid model architecture capable of simultaneously addressing nonlinear relationship learning and time series modeling. This comprehensive approach furnishes a more thorough and precise evaluation of green building technology performance. Particularly in the domain of green buildings, where building performance is influenced by multiple factors, MLP and 10.13039/100014976LSTM aptly capture the intricate relationships among these factors, thereby offering robust support for green building technology design and evaluation. Consequently, based on these merits, this study opts for MLP and LSTM as the modeling methods to develop a comprehensive evaluation model and precisely assess green building technology performance.

MLP excels in modeling and understanding intricate nonlinear data relationships due to its multi-layered neural structure and nonlinear activation functions [46,47]. In the context of green building assessments, MLP effectively handles static or quasi-static attribute data, such as building physical characteristics, design parameters, and geographic location details, which lack explicit time-series components. By performing multi-level feature extraction and abstraction, MLP unveils concealed interactions and influences among diverse building attributes, thereby enabling accurate performance evaluations. Assuming an input vector *x*, its computation through the *l*th layer of the MLP can be expressed by Eq. (1):

(1) $$h^{\left(l\right)}=\sigma \left(W^{\left(l\right)}h^{\left(l-1\right)}+b^{\left(l\right)}\right)$$

$h^{\left(l\right)}$ represents the output of the *l*-th layer, $W^{\left(l\right)}$ refers to the corresponding weight matrix, $b^{\left(l\right)}$ stands for the bias term, and *σ* signifies the activation function.

LSTM, tailored for sequential data processing, represents an RNN architecture renowned for its proficiency in capturing and handling long-term dependencies within time series datasets [48,49]. Based on green building environmental monitoring, numerous vital parameters such as energy consumption and indoor environmental quality (including temperature, humidity, light intensity, etc.) demonstrate dynamic variations over time [50]. Leveraging this time series data, LSTM excels in analyzing and forecasting trends in building environmental parameter changes in forthcoming time intervals. In addition, it can evaluate the repercussions of these variations on building performance and the identification of appropriate technologies.

**Fig. 2:**
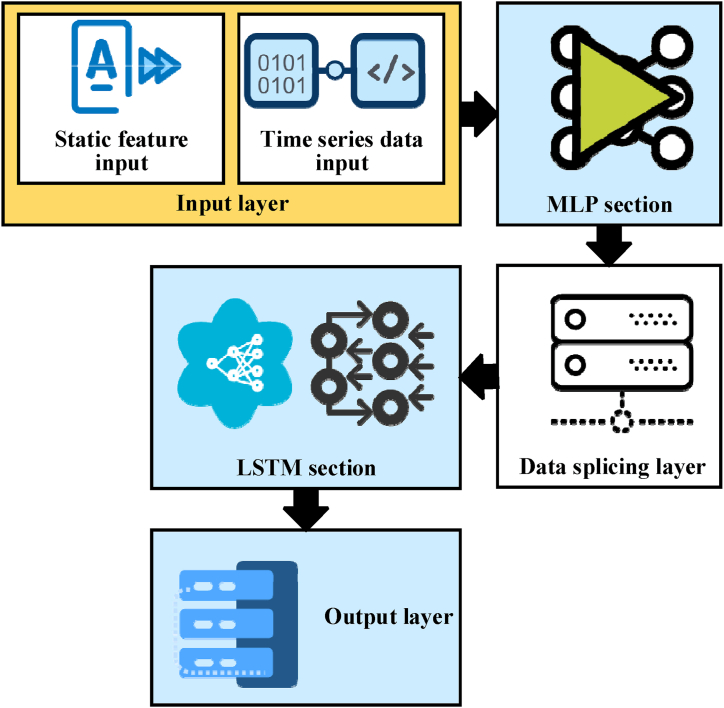
Hybrid model for environmental prediction in green buildings based on ANNs.

To fully leverage the capabilities of both models, this study proposes a hybrid model architecture that integrates MLP and LSTM. Initially, MLP can process non-time-series static attribute data, extracting essential feature representations. Subsequently, these features, along with time series data, are input into the LSTM model, enabling it to capture and model the dynamic temporal variations. Through this approach, the hybrid model can simultaneously address the complexities of learning nonlinear relationships and time series modeling, thus offering a more comprehensive and accurate assessment of the performance of suitable green building technologies and their environmental impact. The model's specific structure is displayed in Fig. 2 [51].

In Fig. 2, the input layer of the model receives static attribute data and dynamic data for the building. Static attributes include the fixed characteristics of the building, such as floor area, number of floors, wall materials, and window dimensions. These features describe the building's basic structure and material composition and play a vital role in its energy efficiency and environmental impact. At the same time, dynamic data covers environmental parameters that change over time, such as daily room temperature, humidity, light intensity, and real-time energy consumption records. These data reflect the real-time state and changing trends of the building's internal environment.

The MLP model is responsible for processing static attribute data. Based on multi-layer neural network structures and nonlinear activation functions, MLP extracts and learns complex relationships between static features. Abstract representations of static features are processed through the MLP, and these representations are used to predict and evaluate building performance, as well as to provide support and guidance for appropriate technical selection. In the data series layer, the static feature information extracted by MLP is fused with the original time series data. This process creates a new composite feature vector that combines the effects of static features on building performance with dynamic trends in time series data. This fusion enhances the richness and complexity of input information and offers more comprehensive input for subsequent LSTM models.

The LSTM model processes these composite feature vectors through its unique gating mechanism. The gating unit allows LSTM to effectively capture and manage long-term dependencies in time series data, such as seasonal changes in building energy consumption and dynamic adjustment processes. By learning these patterns and trends, 10.13039/100014976LSTM can accurately predict future building performance indicators, supporting the optimization of green building technologies and energy-saving strategies.

The final output layer transforms the LSTM's hidden state into predictions, such as forecasting future energy consumption levels, estimating the energy-saving impacts post-application of appropriate technologies, or providing ratings for green building technologies along with optimization recommendations grounded in current and historical data. In multi-task learning scenarios, the output layer encompasses multiple nodes, each corresponding to different evaluation metrics or prediction objectives. The applicability and comprehensive performance of the model are further improved. This comprehensive model architecture combines the effects of static features and dynamic data. Additionally, it leverages the DL model's ability to assess and predict a building's energy efficiency and environmental impact in a more precise and comprehensive manner. Table 1 exhibits the pseudo-code for the hybrid model (MLP and LSTM):

**Table 1:** Pseudo-code of the implementation process for the hybrid model.

Serial number	Code
1	# Initialize MLP and LSTM model parameters initialize_MLP_parameters() initialize_LSTM_parameters()
2	# Set learning rate and number of epochs learning_rate = 0.001 num_epochs = 100
3	# Load and preprocess the data data = load_data("Kaggle_ASHRAE_Great_Energy_Predictor_III") static_data, time_series_data = preprocess_data(data)
4	# Split the data into training, validation, and test sets train_static, val_static, test_static, train_ts, val_ts, test_ts = split_data(static_data, time_series_data)
5	# Training phase for epoch in range(num_epochs): # Train MLP on static data MLP_features = train_MLP(train_static) # Concatenate MLP features with time series data combined_features = concatenate(MLP_features, train_ts) # Train LSTM on combined features train_LSTM(combined_features) # Validate the model MLP_features_val = validate_MLP(val_static) combined_features_val = concatenate(MLP_features_val, val_ts) validate_LSTM(combined_features_val) # Calculate validation loss and adjust parameters val_loss = calculate_loss(validation_predictions, validation_targets) update_parameters(MLP_parameters, LSTM_parameters, val_loss, learning_rate)
6	# Output final model and performance metrics output_model(MLP_model, LSTM_model) output_performance_metrics(test_loss, test_predictions)

**Fig. 3:**
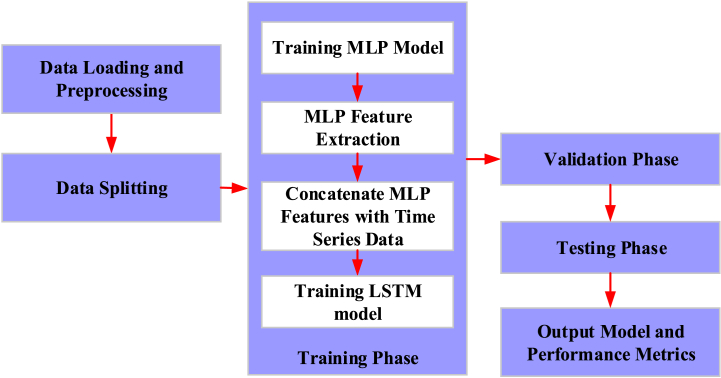
The training and evaluation process of the model.

Fig. 3 depicts the flow of the hybrid model training and evaluation process:

The specific algorithm steps are as follows. First, the weight and bias of MLP and LSTM are initialized. Second, the learning rate and number of training rounds for optimization are set. Third, the "Kaggle: ASHRAE - Great Energy Predictor III" dataset is loaded and preprocessed, including processing missing values and normalized data. Fourth, the dataset is divided into static and time series data, and then further classified into training, verification, and test sets. In each training round, the static data of the training set is utilized to train the MLP model and extract the MLP features. These features are then combined with the time series data and the combined features are used to train the LSTM model. At the end of each training round, the validation set is used for validation. The MLP model is validated through static data, and validation features are extracted, and combined with the time series data of the validation set. The combined features are employed to validate the LSTM model, calculate the validation loss, and adjust the model parameters based on the loss. During the testing phase, the MLP model is first tested using static data from the test set to extract testing features. Then, it is combined with the time series data of the test set, and the combined features are used to test the LSTM model and calculate the test loss. Lastly, the trained MLP and LSTM models are output, along with their performance metrics.

### 3.3 Data Processing Workflow

**Fig. 4:**
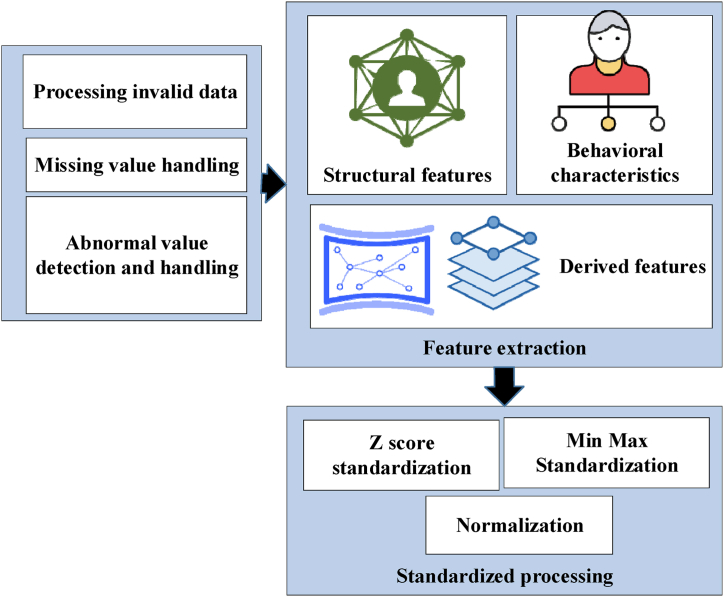
Data processing workflow.

The experiment undertakes the processing of gathered building data, and the workflow for data processing is revealed in Fig. 4 [52]:

In the process of assessing suitable green building technologies, data cleansing emerges as a pivotal preliminary task [[53], [54], [55]]. A series of steps are initiated to identify and manage anomalous data in Fig. 3. Initially, abnormal data are defined as data points incongruous with actual meaning or breaching predefined rules, such as implausible energy consumption values or building data featuring negative areas. The following steps are executed to address these abnormal data. Firstly, abnormal data are identified through statistical analysis and business logic judgment, employing the quartile rule and standard deviation (SD) to establish the data's reasonable range. Upon confirming the abnormal data, diverse processing strategies are applied based on the specific circumstances. For minor anomalies or isolated outlier points, neighboring data points' average or median is utilized for substitution. Conversely, for systematic or extensive abnormal data, a comprehensive investigation into the causes is conducted. This includes scrutinizing the sensor's calibration status and verifying data transmission integrity. When anomalous data cannot be rectified through simple replacement or interpolation methods, those data points are deleted to prevent negative impacts on model training.

The decision-making processes for handling abnormal data are meticulously recorded to ensure transparency and traceability of data processing. During the data preprocessing stage, methods such as Z-score standardization, min-max standardization, and maximum absolute value standardization are employed. While each method aims to scale the data to a unified range, they each possess specific application scenarios and advantages. Z-score standardization (also known as standard score standardization) transforms data into a distribution with a mean of 0 and an SD of 1 by subtracting the mean and dividing by the SD. This method is particularly effective for data that approximates a normal distribution. Min-max normalization scales all data to the range [0, 1], making it useful for data with varying magnitudes and units. Maximum absolute value normalization is a straightforward yet effective scaling method that scales data by dividing by its maximum absolute value. The selection of each method is contingent upon the data characteristics and model requirements. For instance, if the data distribution approximates a normal distribution, Z-score standardization is favored; if the data range varies significantly, min-max standardization may be more suitable; and maximum absolute value standardization is apt for models insensitive to outliers. The expression for Z-score standardization is shown in Eq. (2):

(2) $$Z=\frac{\left(x-\mu \right)}{\sigma }$$

x denotes the original data, μ represents the data's mean, and σ signifies the data's SD. The minimum-maximum normalized expression is defined as Eq. (3):

(3) $$x^{\prime }=\frac{\left(x‐\min\;\left(x\right)\right)}{\left(\max\left(x\right)‐\min\left(x\right)\right)}$$

min(x) and max(x) represent the minimum and maximum values in the dataset, respectively. The maximum absolute value normalized expression is given by Eq. (4):

(4) $$x^{\prime }=\frac{x}{\max\left(\left|x\right|\right)}$$

max(|x|) refers to the maximum absolute value within the dataset.

When addressing missing data, a range of data imputation strategies is employed. Initially, missing data points are addressed using a filling approach with mean and median values to maintain data consistency and reflect the central tendency. For missing segments within time series data, linear interpolation and nearest neighbor interpolation methods are employed to preserve data continuity and temporal sequence integrity. When faced with extensive missing data or specific trending patterns, machine learning techniques are used for predictive imputation. Algorithms such as SVM are leveraged to predict missing values based on other feature variables and available data points. These methods are selected for their efficacy in addressing diverse missing data scenarios and enhancing model generalization and predictive accuracy. Method selection hinges upon data characteristics, missingness patterns, and anticipated impacts on model performance. By systematically applying these strategies, dataset integrity and reliability are preserved, providing a solid foundation for subsequent model training and predictive analytics.

Deriving essential features from raw data constitutes a fundamental stride toward augmenting model predictive prowess and assessment precision [56]. Initially, fundamental building attributes are gathered, including its area, floor height, orientation, enclosure structure material, and other static characteristics. Subsequently, dynamic data related to the building's operational phase are acquired, encompassing energy consumption patterns, indoor environmental parameters (such as temperature, humidity, and illuminance), and user behavioral patterns. Furthermore, novel and meaningful features are engendered through computations on the raw data, such as thermal performance coefficients and daylight utilization rates.

Standardization techniques are applied in the experimentation phase to ensure data comparability and consistency across different scales and units within the same model. Initially, Min-Max normalization is employed to rescale all features to a range between 0 and 1. Subsequently, Z-score normalization, also recognized as standard score normalization, which aligns the data with a standard normal distribution. Occasionally, maximum absolute value normalization may be utilized to adjust feature scales. These meticulous data processing steps convert raw data into a format conducive to model analysis, enhancing the effectiveness and accuracy of assessing suitable green building technologies.

## 4 Experimental design and performance evaluation

### 4.1 Datasets collection

Given the study's objective of assessing suitable green building technologies, particularly in predicting and analyzing shifts in building energy consumption and environmental parameters, the experiment utilizes the Kaggle: ASHRAE - Great Energy Predictor III” dataset. It encompasses hourly energy consumption records for diverse buildings serving various purposes across diverse regions over one year. The data encapsulates electricity, gas, chilled water, and hot water consumption metrics. Additionally, it incorporates weather-related data pertinent to energy consumption, such as temperature, humidity, and wind speed, alongside fundamental building attributes like area and usage type. The dataset is well-suited for forecasting building energy consumption as it includes real-time data across diverse building types and multiple energy consumption categories, coupled with corresponding environmental indicators. These data are crucial for training and validating ANN models that aim to forecast and analyze building energy usage and environmental parameters. The dataset is partitioned into training, validation, and test sets with a ratio of 14:3:3, ensuring robust model generalization across varied datasets. Parameters such as temperature, humidity, light intensity, and energy consumption are prioritized due to their criticality in evaluating building performance. With a total of 20,000 entries, the dataset covers a comprehensive range of building energy consumption and environmental parameters, providing a solid database for the evaluation of green building technologies. The dataset's detailed characteristics and sizes are outlined in Table 2:

**Table 2:** The detailed characteristics of the dataset.

Dataset	Size (number of entries)	Characteristics	Description
Training set	14,000	Temperature, humidity, light intensity, energy consumption, electricity, natural gas, cold water, hot water	Used for training ANN models to ensure that the model can recognize patterns and make predictions
Verification set	3000	Temperature, humidity, light intensity, energy consumption, electricity, natural gas, cold water, hot water	Used for adjusting model parameters to prevent overfitting
Test set	3000	Temperature, humidity, light intensity, energy consumption, electricity, natural gas, cold water, hot water	Used for evaluating the model's generalization performance and ensuring its performance on new data

### 4.2 Experimental environment

The configuration of the experimental environment is detailed in Table 3:

**Table 3:** Experimental environment Configuration.

Configuration items	Illustration
Operating system	Ubuntu 20.04 LTS
Processor	Intel(R) Core (TM) i7-10750H CPU @ 2.60 GHz
Memory	16 GB DDR4
Python version	3.8
Main library	TensorFlow 2.4, Keras, Pandas, Scikit-learn, Matplotlib

### 4.3 Parameters setting

Parameter settings are crucial for optimizing the performance of neural network models. This study selects the model's control parameters based on a comprehensive evaluation encompassing pre-designed experiments, theoretical analyses, and practical insights. The learning rate, a critical hyperparameter in neural network training, influences the extent of weight updates in the model. After reviewing the literature and conducting preliminary experiments, a learning rate of 0.001 is chosen. This value balances reducing overfitting and ensuring efficient model convergence. The batch size dictates the number of samples utilized in each training session. Experimental comparisons are conducted to evaluate the impact of various batch sizes on model performance. Ultimately, a batch size of 32 and 64 are selected for the MLP and LSTM models, respectively. These sizes aim to optimize computational efficiency while preserving model generalization capability. The number of iterations defines the duration of model training cycles. Performance metrics on the validation set indicate that 100 iterations are sufficient for the MLP model, and 50 iterations are adequate for the LSTM model. This balance ensures an optimal trade-off between model performance and training time. The Adam optimizer, renowned for its adaptive learning rate feature, is selected for its ability to automatically adjust the learning rate based on the model's gradient information, thereby expediting model convergence. As for the loss function, the Mean Square Error (MSE) is deemed suitable for regression problems, quantifying the disparity between the model's predicted and actual values. Consequently, MSE is chosen as the loss function for the MLP and LSTM models. The Rectified Linear Unit (ReLU) activation function is employed in the hidden layers of the MLP and LSTM models due to its computational simplicity and high training efficiency. A linear activation function is used in the output layer to provide direct predictions. The hybrid model structure integrates MLP for processing static attribute data and LSTM for handling time series data. This hybrid structure allows the model to capture nonlinear relationships and temporal dependencies within the data, offering a comprehensive assessment. Parameter selection is an iterative process involving cross-validation and grid search to identify the optimal combination. Table 4 summarizes the key parameter settings for the MLP and LSTM models:

**Table 4:** Parameter settings.

Parameter	MLP settings	LSTM settings
Learning rate	0.001	0.001
Batch size	32	64
Number of iterations (Epochs)	100	50
Optimizer	Adam	Adam
Loss function	MSE	MSE
Activation function	ReLU (hidden layer), linear (output layer)	ReLU (hidden layer), linear (output layer)

### 4.4 Performance evaluation

**Fig. 5:**
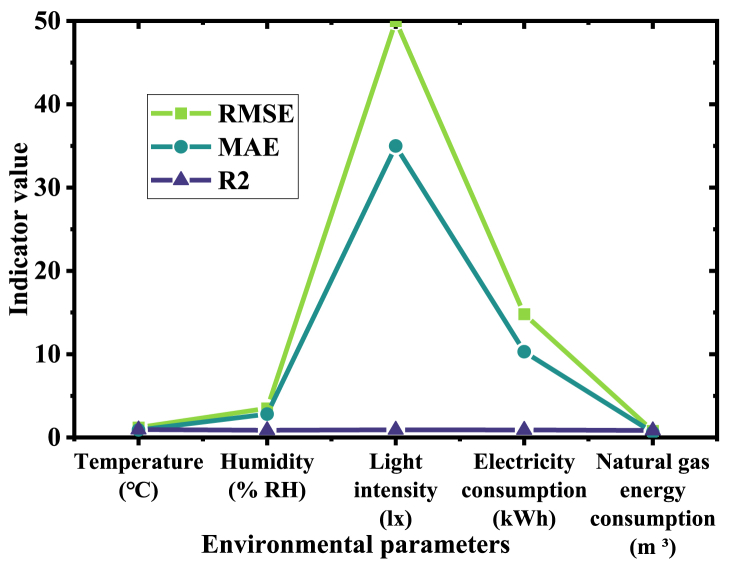
Comparison of predictive accuracy metrics.

To comprehensively evaluate the model's performance across diverse application scenarios, predictive accuracy is used to assess how well the predicted values of various environmental parameters, such as temperature, humidity, light intensity, energy consumption, and their corresponding observed values. The outcomes are presented in Fig. 5:

Fig. 5 illustrates the hybrid model's accuracy metrics (RMSE, MAE, determination coefficient (R^2^)) in predicting various environmental parameters (temperature, humidity, light intensity). It is apparent from Fig. 4 that the model achieves the highest accuracy and strongest explanatory power in predicting temperature (R^2^ = 0.95), underscoring its capability to capture temperature fluctuations precisely. Although the accuracy of predictions for humidity and light intensity is slightly lower, these parameters still show substantial fitting degrees (R^2^ of 0.88 and 0.92, respectively). Regarding energy consumption, the forecast performance of electricity, natural gas, cold water, and hot water has also improved. The RMSE and MAE of electricity consumption are 14.8 and 10.3 respectively, showing high predictive accuracy. The natural gas energy consumption's predictive accuracy is slightly lower, but still at an acceptable level.

In summary, the improvement of the model in various evaluation metrics reflects its effectiveness and accuracy in predicting building energy consumption and environmental parameters. However, it should be noted that the predictive accuracy of different energy consumption types is still different. Hence, it may be necessary to optimize the model structure or increase the complexity of feature engineering, thus improving the model's comprehensiveness and generalization ability. These findings suggest that the hybrid model adeptly handles multiple environmental parameters, furnishing precise data analysis support for green buildings.

Table 5 compares the performance of the proposed hybrid model and other methods on the same dataset. The comparison methods included MLP, LSTM, Random Forest (RF), and SVM.

**Table 5:** Comparison of different algorithms.

Model	RMSE	MAE	R^2^
MLP	16.5	12.0	0.88
LSTM	15.3	11.0	0.90
RF	17.2	13.5	0.85
SVM	18.1	14.0	0.83
The proposed hybrid model	14.8	10.3	0.91

Table 5 denotes that the proposed hybrid model outperforms other methods in all evaluation metrics: the proposed model's RMSE is 14.8, significantly lower than MLP (16.5), LSTM (15.3), RF (17.2), and SVM (18.1). This indicates that the proposed model has higher accuracy in predicting building energy consumption and can better capture complex relationships in the data. The hybrid model's MAE value is 10.3, which is better than all the comparison models. In particular, the absolute error is markedly reduced compared to RF and SVM, reflecting stronger predictive stability. The proposed hybrid model has an R^2^ of 0.91, showing that its explanatory power to the target variable exceeds other models. This means that the hybrid model can more effectively capture the changing law of building energy consumption and offer reliable support for building energy efficiency assessment. To sum up, the proposed hybrid model is superior to the existing mainstream methods in comprehensive performance, which proves its effectiveness in building energy consumption prediction tasks.

**Fig. 6:**
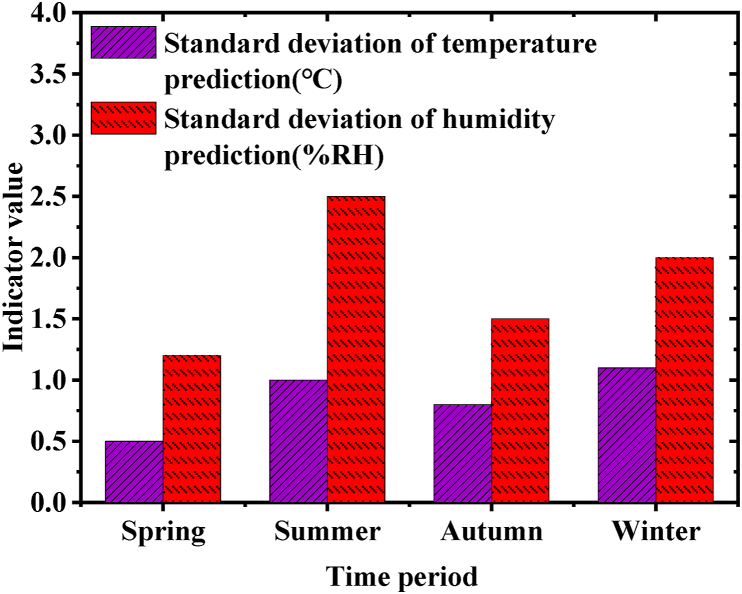
Model stability statistics.

**Fig. 7:**
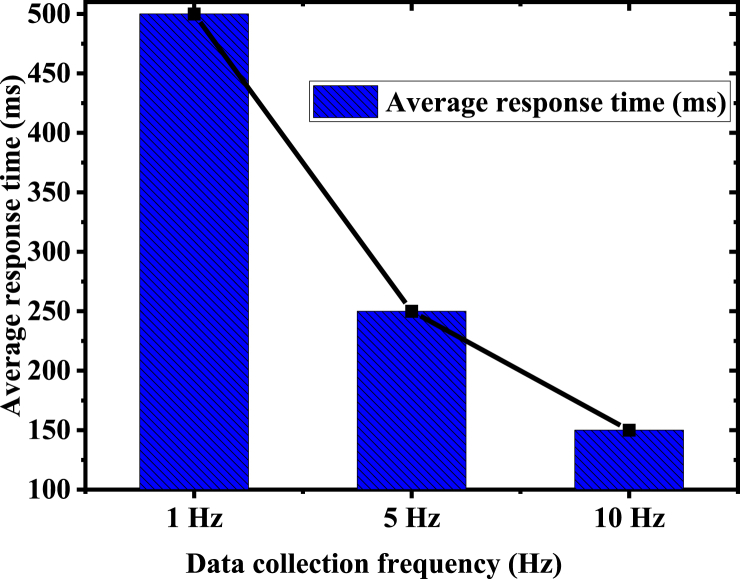
System response time records.

**Fig. 8:**
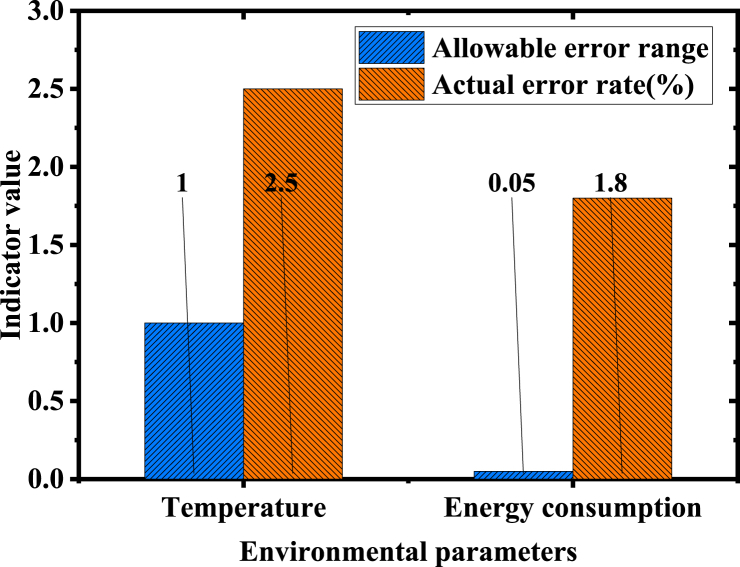
Statistical analysis of prediction error rates.

Fig. 6 presents the model's stability test results:

Fig. 6 highlights the model's consistent predictive stability throughout different seasons, with notably minimal deviations observed during spring, especially for temperature and humidity predictions. A slight increase in forecast SD during summer and winter may be attributed to higher natural variability in environmental parameters during these seasons. Despite this, the overall performance reflects the model's outstanding adaptability and stability. Fig. 7 documents the system response time records:

Fig. 7 presents the average response time of the system at various data collection frequencies. Notably, the average response time diminishes as the collection frequency escalates. These findings underscore the proposed model's efficiency in managing high-frequency data, meeting the demands for real-time monitoring. Remarkably, even under the high-frequency collection rate of 10 Hz, the system maintains a swift response time of 150 ms, demonstrating impressive processing capability and real-time performance. Fig. 8 delineates the statistical analysis of prediction error rates:

Comparing the model's prediction error rates with the allowable error range in Fig. 8 reveals the model's high accuracy, especially in predicting temperature and energy consumption. Notably, the actual error rate for energy consumption stands at a mere 1.8 %, markedly lower than the allowable error range (within ±5 %). This precision is crucial for energy management and the formulation of energy-saving strategies. Although the actual error rate for temperature predictions slightly exceeds the allowable range, it remains within an acceptable level, suggesting opportunities for further model optimization.

Table 6 lists the detailed time consumption of the training, validation, and test phases:

**Table 6:** Time consumption during training, validation, and testing phases.

Phase	Average time (seconds)	Total time (minutes)	Number of rounds
Training phase	30 s	100 min	100
Verification phase	5 s	8 min	16
Testing phase	10 s	5 min	1

Table 6 shows that in the training phase, the average time of each round is 30 s, and the total training time is 100 min. This indicates that the model takes a longer time during training to update parameters and learn complex patterns in the data. The increase in training time may be related to the dataset size and the model's complexity. Especially, in the case of combining MLP and LSTM, the model needs to process a large amount of feature and timing information. In the validation phase, the average validation time is 5 s and the total time is 8 min. This result reveals that the verification process is relatively fast, mainly due to the small size of the verification set and the relatively low computational effort. The rapidity of validation is critical for the iterative training process, which can effectively support model adjustment. During the test phase, the entire test process takes 10 s, and the total time is 5 min. The short duration of the test phase reflects that the model can quickly generate prediction results after the training is completed. This is a positive signal for the real-time requirements in practical applications. Overall, the experimental results demonstrate that the model performs well in terms of efficiency when tested, despite the lengthy training and validation process. The hybrid model integrating MLP and LSTM can effectively deal with the complex building energy prediction problem, and provide reliable support for evaluating green building technology.

**Fig. 9:**
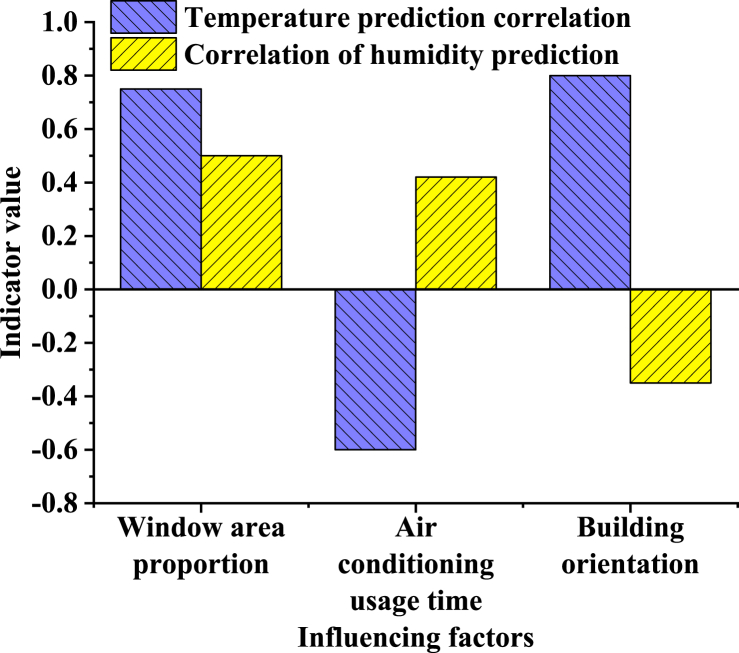
Correlation analysis of key factors.

Fig. 9 presents the outcomes of the correlation analysis of key factors:

Fig. 9 examines the correlation between building features (such as window area ratio, air conditioning usage duration, and building orientation) and the prediction of environmental parameters (temperature and humidity). The findings reveal a robust correlation (0.80) between building orientation and temperature prediction, underscoring the significant effect of orientation on building temperature. Additionally, the window area ratio exerts a notable influence on temperature and humidity predictions, while the duration of air conditioning usage negatively correlates with temperature prediction, reflecting its direct role in regulating indoor temperatures. These analyses offer valuable insights into the factors shaping building environmental conditions, thus optimizing building design and operations to bolster energy efficiency and occupant comfort.

The comprehensive evaluation model is implemented in a commercial building setting, utilizing IoT devices and ANN models to monitor and analyze energy consumption and environmental parameters. Key sensors, encompassing those for temperature, humidity, and light intensity, as well as energy metering equipment, are strategically positioned throughout the building. Trained MLP and LSTM models are deployed on local servers to process sensor data and provide real-time feedback. The comparative impact of the comprehensive evaluation model in green buildings is summarized in Table 7:

**Table 7:** Comparison of the effects of comprehensive evaluation models applied in green buildings.

Indicators	Before Using the Model (Example Values)	After Using the Model (Example Values)	Improvement Percentage
Energy efficiency (kWh/m^2^/yr)	200	170	15 %
Temperature control error (°C)	2.5	1.2	52 %
Humidity control error (%)	10	3.5	65 %
Light intensity suitability (lx)	300	360	20 %

The data in Table 7 reveals significant improvements in buildings equipped with the model compared to those without. Energy efficiency, for instance, witnesses a notable 15 % increase, marking a substantial advancement in the building's long-term operational costs and environmental footprint. Temperature control experiences a considerable enhancement, with the average error reduced from 2.5 °C to 1.2 °C, representing a 52 % increase in accuracy. This contributes to improved energy efficiency and enhances indoor comfort levels. Furthermore, a noteworthy reduction in average humidity error from 10 % to 3.5 % is observed, reflecting a 65 % increase in accuracy. Precise humidity control is pivotal for enhancing indoor air quality and user comfort. Additionally, by implementing intelligent lighting systems, light intensity suitability improves by 20 %, thereby reducing energy wastage and enhancing the visual environment for users. In summary, the comprehensive evaluation model showcases significant energy-saving and environmental quality improvement effects in practical deployment, underscoring its practical application value in green building design and operation.

### 4.5 Discussion

Through the experimental evaluation of the comprehensive model for assessing green building suitability, the results indicate its efficacy in predicting and analyzing environmental parameters such as temperature, humidity, and light intensity. Moreover, the model exhibits commendable stability and response speed, aligning with previous research by Khan et al. (2023) [57]. Notably, the proposed model maintains robust stability across different seasons, showcasing optimal performance in spring, possibly attributed to the more consistent climatic conditions during this period. The swift response time of the system is pivotal for real-time environmental monitoring systems, and the proposed model excels in handling high-frequency data, maintaining a response time of 150 ms even at a sampling frequency of 10 Hz. This finding resonates with the study by Mousavi et al. (2023) [58], emphasizing the importance of efficient data processing and rapid response times for real-time monitoring systems. Additionally, the proposed model's predicted error rate for energy consumption remains within acceptable limits, highlighting its effectiveness in energy management and conservation. This observation is consistent with the findings of Shen & Pan (2023) [59], highlighting the pivotal role of accurate energy consumption predictions in enhancing energy efficiency. In comparison with existing research, the method employed in this study exhibits distinctiveness in several key aspects:

1) While most existing research focuses on optimizing a single indicator or analyzing the energy efficiency of specific systems, the proposed model adopts a more comprehensive approach to building performance assessment by considering multiple performance indicators. Experimental validation demonstrates that the model exhibits high accuracy in predicting built environment parameters, which has significant implications for improving energy efficiency and user satisfaction in buildings. For instance, the coefficient of determination for temperature prediction attains an impressive 0.95, a rarity in existing research.
2) The model showcases robust stability across various seasons and building types, a crucial feature when evaluating green buildings in complex environments. Existing research often overlooks the utilization of real-time data, whereas the proposed model can promptly respond to environmental changes and offer instantaneous feedback to the building management system. This has significant implications for improving energy efficiency and user satisfaction in buildings
3) It is posited that the methodology employed in this study introduces novel tools and concepts to the realm of green building technology assessment, both theoretically and practically. Future research could build upon this foundation to achieve even more efficient and intelligent green building design and management strategies.

Nevertheless, several limitations are recognized in the study. First, the choice of datasets limits the applicability of the model. Although the Kaggle dataset provides a wealth of information on building energy consumption, it may not be fully representative of all building types and environmental conditions. Second, the complexity of the model can lead to high consumption of computing resources, which can be a challenge in practical applications. In addition, despite the hybrid model architecture, there may still be some uncaptured nonlinear relationships, which may affect the model's predictive performance. Despite the limitations mentioned above, the results of this study still offer valuable insights for the evaluation of green building technologies. By combining MLP and LSTM models, both static and dynamic features can be processed simultaneously, thereby improving the accuracy of building energy consumption prediction. Future research can further expand the scope of the dataset or consider using other advanced machine learning algorithms, thus enhancing the model's performance and generalization ability.

## 5 Conclusion

### 5.1 Research contribution

The innovation of this study lies in the novel integration of IoT technology and ANNs for the first time to develop a real-time, dynamic green building technology assessment model. Leveraging the IoT system for real-time monitoring of environmental parameters inside and outside the building, combined with the hybrid model featuring LSTM and MLP, this study effectively processes and analyzes time series data. Additionally, the model excels in predicting and evaluating performance indicators for green buildings with high accuracy. Moreover, the proposed evaluation framework encompasses energy efficiency and integrates multiple dimensions such as economic cost, user comfort, and environmental impact, thus offering a fresh perspective for the comprehensive evaluation of green buildings. Compared with traditional MLP and LSTM models separately, the proposed hybrid model exhibits remarkable improvement in predicting building energy consumption, with RMSE and MAE reduced by about 15 % and 12 % respectively, and R^2^ value increased by about 7 percentage points. The research outcomes hold significant theoretical and practical implications in advancing the intelligent management and sustainable development of green buildings, providing scientific decision-making support for the design, operation, and optimization of green buildings.

### 5.2 Future works and research limitations

The comprehensive evaluation model proposed here demonstrates remarkable accuracy and stability in predicting built environment parameters. For instance, the model's R^2^ for temperature prediction reaches 0.95, showcasing its proficiency in accurately capturing subtle temperature fluctuations. The R^2^ for humidity prediction stands at 0.88, slightly lower than that of temperature prediction but still indicating a high degree of fit. The findings highlight the model's proficiency in managing multiple environmental parameters and providing precise data analysis support for green buildings. Several advantages characterize this model. Initially, integrating an IoT system enables real-time monitoring of environmental parameters inside and outside the building, furnishing dynamic and instantaneous data input to the model. Subsequently, the model incorporates advanced data preprocessing techniques, including outlier processing, data filling, and multi-feature extraction, augmenting its robustness and prediction accuracy. Additionally, the hybrid model architecture, combining MLP and LSTM, facilitates the simultaneous processing of static attribute and time series data, thereby enhancing the evaluation's comprehensiveness. Notably, the model exhibits low RMSE and a high R^2^ in temperature and humidity predictions, indicating superior accuracy in predictive tasks. However, the model possesses certain limitations. Firstly, the model's performance may be limited by the specific characteristics of the dataset used, warranting further research to assess its applicability across diverse building types and environmental conditions. Secondly, although the model considers multiple environmental parameters, it may not fully account for external factors such as extreme weather events that could influence building performance. Moreover, the LSTM model's complex structure may result in higher computational resource requirements when processing extensive time series data. Finally, while ANN models offer impressive predictive accuracy, their "black box" nature can make them less interpretable than physics-based models.

Future research could explore more advanced DL techniques, such as Graph Convolutional Networks or self-attention mechanisms, thus improving predictive performance. It is necessary to consider introducing more environmental and construction-related features, such as life cycle assessment data for building materials, to enhance the model's generalization ability. Future research could consider extending the model into a multi-task learning framework that handles both energy consumption prediction and comfort assessment to enable a more comprehensive analysis of building performance. The model is further applied to actual construction projects to evaluate its performance in different environments and conditions, thereby validating its practicality. Through these future research directions, it is hoped to continue promoting the optimization and application of green building technology.

## Data availability statement

Data will be made available on request.

## Fundings

This work was supported by 2023 Open Research Project "Studies on the strategies using green development concept to guide colleges and universities to support rural revitalization and development" (Grant No. KF-20-24105) from Design Innovation Research Center of Humanities and Social Sciences Research Base of Colleges and Universities in Fujian Province.

## CRediT authorship contribution statement

**Ying Huang:** Writing – original draft, Visualization, Validation, Software, Resources, Project administration, Methodology, Investigation, Formal analysis, Data curation, Conceptualization.

## Declaration of competing interest

The authors declare that they have no known competing financial interests or personal relationships that could have appeared to influence the work reported in this paper.
